# Mechanical power of ventilation and driving pressure: two undervalued parameters for pre extracorporeal membrane oxygenation ventilation and during daily management?

**DOI:** 10.1186/s13054-023-04375-z

**Published:** 2023-03-14

**Authors:** K. Hoppe, E. Khan, P. Meybohm, T. Riese

**Affiliations:** University Hospital Würzburg, Department of Anaesthesiology, Intensive Care, Emergency and Pain Medicine, Oberdürrbacher Str. 6, 97080 Würzburg, Germany

**Keywords:** ARDS, Ventilation, ECMO indication, Mechanical power, Driving pressure

## Abstract

The current ARDS guidelines highly recommend lung protective ventilation which include plateau pressure (*P*_plat_ < 30 cm H_2_O), positive end expiratory pressure (PEEP > 5 cm H_2_O) and tidal volume (*V*_t_ of 6 ml/kg) of predicted body weight. In contrast, the ELSO guidelines suggest the evaluation of an indication of veno-venous extracorporeal membrane oxygenation (ECMO) due to hypoxemic or hypercapnic respiratory failure or as bridge to lung transplantation. Finally, these recommendations remain a wide range of scope of interpretation. However, particularly patients with moderate-severe to severe ARDS might benefit from strict adherence to lung protective ventilation strategies. Subsequently, we discuss whether extended physiological ventilation parameter analysis might be relevant for indication of ECMO support and can be implemented during the daily routine evaluation of ARDS patients. Particularly, this viewpoint focus on driving pressure and mechanical power.

## Why extended monitoring of pre-ECMO ventilation and indication might be relevant?

Several studies analyzed the potential survival benefit of extracorporeal membrane oxygenation (ECMO) in severe SARS-CoV 2 infected patients. However, the results were highly variable. International registries reported in-hospital mortality rates 90 days after initiation of ECMO therapy of 38% which is in range with the pre-SARS-CoV 2 era data [1, 2]. Recently, Whebell et al. [3] reported a very low in hospital mortality rate of 24% and an absolute reduction of mortality in the ECMO-treated patient group after propensity score matching. In contrast, others analyzed large patient cohorts encompassing 243 or 673 patients and revealed overall in hospital mortality rates up to 68% in the ECMO treated patient collective [4, 5]. Indeed, these differences might be ascribed to center experience and patient specific co-morbidities particularly age or prior immunosuppressive therapy [5]. However, the differences in outcome might also be explained by different center specific indications of ECMO support and ventilation invasiveness pre implantation. One first hint might be given by a detailed analysis of the EOLIA trial. The majority of inclusions (82%) were due to hypoxemia and the reported 60 day mortality rate was 35% in the ECMO-treated patients and 46% in the control group [6]. However, the reported mortality of the patient group who were included due to refractory acidosis by compromised protective ventilation the 60 day mortality was lower (24% in the ECMO group versus 55% in the control group) [6, 7].

**Fig. 1:**
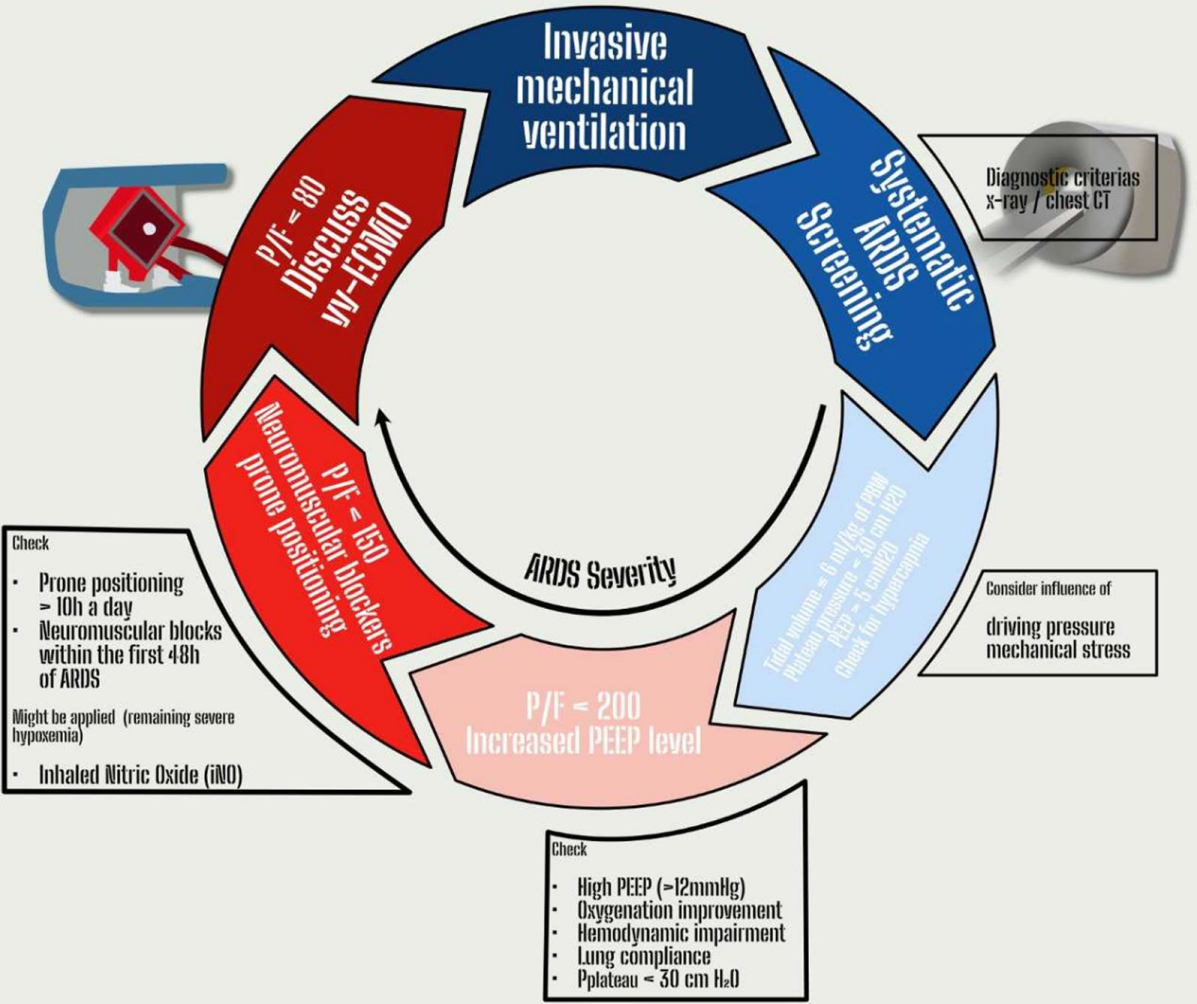
Current guidelines for ARDS treatment [8]

The ELSO guidelines recommend veno-venous ECMO implementation after exclusion of contraindications in the following circumstances: (i) hypoxemic respiratory failure (PaO_2_/FiO_2_ < 80 mm Hg) after optimal medical management including prone positioning trial; (ii) hypercapnic respiratory failure ( pH< 7.25), despite optimal conventional mechanical ventilation (respiratory rate 35 bpm and plateau pressure ≤ 30 cm H_2_O) (iii) ventilatory support as a bridge to lung transplantation or primary graft dysfunction following lung transplant (Fig. 1) [8]. Conclusively, these recommendations leave plenty of room for personal interpretation.

In this viewpoint, we summarize the relevance of the current recommendations for acute respiratory distress syndrome (ARDS) and discuss whether extended physiological ventilation parameter analysis might improve patients` outcome. In particular, we explain the physiological and clinical relevance of driving pressure and mechanical power ventilation.

### Current background of lung protective ARDS ventilation

In 1998 Dreyfuss et al. [9] reported by application of an experimental animal model, that high inflation pressure due to ventilation with high tidal volumes results in increased pulmonary edema and was reduced by straps around the chest and abdomen [10]. Apart from the avoidance of high volume ventilation, the application of positive end exspiratory pressure (PEEP) of 10 cm H_2_0 reduced peri-vascular and alveolar edema reflecting the high relevance between over-distension and low end-expiratory lung volume for ventilator-induced lung injury (VILI) [10, 11]. These findings were approved in large human clinical trials and are the basis of the current ARDS guidelines which subsequently recommend *P*_plat_ < 30 cmH_2_O, PEEP > 5 cmH_2_O and *V*_t_ 6 ml/kg of predicted body weight (BPW) [Fig. 1] [13]. However, the Lung safe study prospectively analyzed more than 29,000 patients in 50 countries and revealed sub-optimal application of lung protective ventilation [12]. Particularly, *P*_plat_ was considered in only 40% of the ARDS patients and of these only two-thirds were ventilated in a lung protective mode (tidal volume ≤ 8 ml/kg BPW and *P*_plat_ ≤ 30 cmH_2_O) [12, 13]. Indeed, it is not surprising, that these single values might not be adequate for all ARDS patients. While some patients suffer severe carbon dioxide retention at *V*_t_ ventilation, others may be intolerant to high PEEP levels due to circulatory insufficiency or do not benefit from increased PEEP due to limited recruitability. Otherwise, inexactitude realization of lung protective ventilation might have serious consequences particularly for the patients with moderate-severe to severe ARDS. Lung lesions are distributed unequally and injured lung tissue or atelectasis coexists with aerated or normal lung tissue [14]. This is accompanied by a marked heterogeneity in ventilation. Particularly within the border areas between aerated and atelectatic regions up to four to five times increased stretching forces were suggested by a mathematical model application [15]. Subsequently, the potentially injurious ventilator settings were applied to a progressively smaller and more inhomogeneous “baby lung”.

### What does driving pressure stand for and why might it be clinically relevant?

The applied pressure to support the delivery of the *V*_t_ is defined as driving pressure, which represents the strain applied to the lung during each ventilatory cycle. Driving pressure comprises the difference between the airway pressure at the end of the inspiration (*P*_plateau_) and PEEP [16–18]. The quotient between *V*_t_ and driving pressure represents the static compliance of the respiratory system. Finally, the driving pressure reflects the *V*_t_ in relation to the compliance of the respiratory system (*C*_RS_) which is associated with ARDS severity as it reflects the proportion of lung availability for ventilation. In patients suffering ARDS, C_RS_ was reported to be directly related to lung functional size [19–21].

However, the clinical benefit of moving a mere *V*_t_ to a *C*_RS_-based ventilation strategy is currently discussed. Amato et al. [22] suggested that the driving pressure was strongly associated with mortality and a decrease due to changed ventilator settings was associated with improved survival. Interestingly, this correlation was also persistent during lung protective ventilation. Recently Haudbourg et al. [19] reported that a driving pressure guided ventilation strategy with target levels between 12 and 14 cm H_2_O required *V*_t_ adoptions in 90% of the patients. In contrast, earlier reports suggested no significant advantage of the driving pressure concept compared to the *P*_plateau_ in view of mortality [13, 23]. However, available data is very limited and based on retrospective and observational designs or with very limited encompassed patients. Moreover, the transpulmonary driving pressure (the difference between *P*_plateau_ minus PEEP and *P*_esophageal-Plateau_ minus *P*_end-expiratory oesophageal_) which particularly includes chest wall elastance was reported to better reflect lung stress [16, 24]. Finally, VILI was suggested to be triggered by mechanical stress and strain which is determined by *V*_t_ and endexspiratory lung volume (corresponding to higher respiratory system elastance)—both parameters are represented by the driving pressure [25]. However, driving pressure is physiologically and mathematically coupled with *V*_t_, elastance and subsequent disease severity [26, 27]. Therefore Goligher et al. [26] analyzed the relationship between the respiratory elastance and mortality for the higher and lower *V*_t_ strategy arms. The absolute risk reduction associated with a lower *V*_t_ ventilation strategy increased progressively with increased elastance. In conclusion, driving pressure should be monitored during daily routine practice in ARDS patients and critically evaluated for *V*_t_ reductions below 6 ml/kg PBW when exceeding 15 cm H_2_O. Of course, the threshold is currently a matter of debate and remains to be evaluated within clinical trials. In this regard, clinicians have to be aware that PEEP changes might subsequent influence elastance (increase: overdistension; decrease: lung recruitment). Finally, clinical trials which evaluate the elastance based on very low *V*_t_ ventilation strategies potentially facilitated by extracorporeal CO_2_ removal strategies are urgently needed to optimize lung protection in ARDS patients.

### What does  mechanical power stand for and why might it be clinically relevant?

**Fig. 2:**
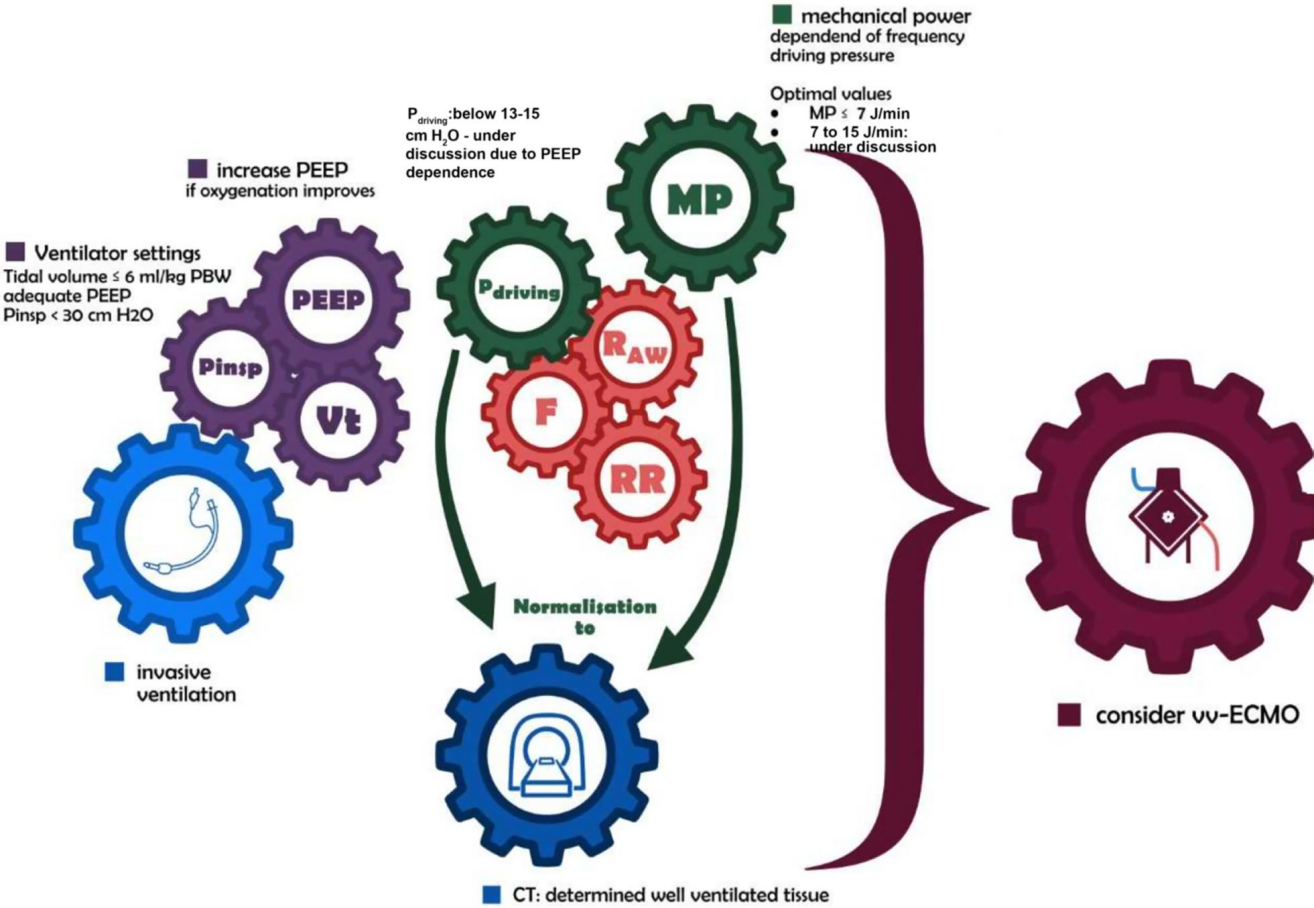
Individualized and normalized mechanical power-based and driving pressure-based ventilator management: Monitoring static ventilation parameter (*P*_insp_, PEEP and *V*_t_) (purple gear) at least twice a day is highly recommended. If *P*_driving_ exceeds 15 cm H_2_O, *V*_t_ reductions below 6 ml/kg PBW and PEEP reductions should be critically evaluated. For in-depth analysis MP should be determined (Fig. 3). While values below 7 J/min were reported to be save, values between 7 to 15 J/min are currently a matter of debate. MP is a function of its components (*V*_t_, *P*_plateau_, inspiratory Flow, RR and PEEP) and every single parameter should be evaluated for potential reductions (red gear) [40]. Normalization to the CT determined well ventilated lung tissue is highly recommended. If sufficient oxygenation and adequate CO_2_ removal is not realizable after optimization of the ventilator strategies, indication for ECMO and extracorporeal CO_2_ removal devices should be critically evaluated

**Fig. 3:**
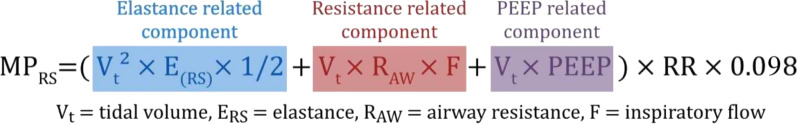
Extended formula for PCV ventilation [33, 52]

While the relevance of the static ventilation parameter including *V*_t_, PEEP, *P*_plateau_ and driving pressure is well established, current increasing evidence suggests a relevant contribution of the dynamic ventilation parameter including respiratory rate, inspiratory and expiratory airflow to VILI (Fig. 2) [27, 28]. Subsequently, the concept of mechanical power defined as the product of respiratory rate and total inflation energy gained attention for ventilation monitoring. Inflation energy comprises three components: (i) the power to overcome airway resistance during gas movements; (ii) the power to inflate the lung and chest wall movements, and (iii) the power to overcome end-exspiratory pressure-related recoil of the lung and respiratory system (Fig. 3) [29]. When these parameters are multiplied by the respiratory rate, mechanical power applied to the respiratory system per minute results [24]. Particularly the respiratory rate is an undervalued parameter during clinical practice. However, recent evidence revealed two ventilation strategies (High *V*_t_; *P*_Plateau_ 34 cm H_2_O; driving pressure 29 cm H_2_O versus RR 40 pb; *P*_Plateau_ 17 cm H_2_O; driving pressure 9 cm H_2_O) caused the same degree of lung lesion after 48 h which suggests that also increased respiratory rate might cause increase lung injury in a specific damaged area [28, 30].

Neto et al. [31] analyzed 8207 patients receiving invasive ventilation for at least 48 h hours and suggested that mechanical power in the second 25 h of ventilation might be independently associated with increased mortality of critically ill patients, a lower number of ventilator free days and survival at day 28. Concordantly, Umer et al. [32] reported that the cumulative exposure to higher intensities of mechanical ventilation was harmful and that a significant increase in the hazard of death was found to be associated with each daily increment in driving pressure and mechanical power. In contrast, Coppola et al. reported that mechanical power resulting from airway pressure and from transpulmonary pressure were assessed and not related to the outcome of ARDS patients [33]. However, both the normalization to compliance and to well-inflated tissue independently increased the intensive care mortality of 1.78 and 2.64 times for one unit increase [33].

An experimental animal setting confirmed that high mechanical power ventilation is associated with increased levels of interleukin 6 and amphiregulin expression and correlated well with diffuse alveolar damage score and club cell protein 16 extression [34]. Importantly, mechanical power combines the effects of different variables and changing of none variable may not necessarily protect the lungs, as it may increase mechanical power delivered to the lung [35]. In detail, a decreased *V*_t_ might not necessarily be translated into lung protection if respiratory rate is increased for compensation or PEEP increases may not be protective if not accompanied by declined driving pressure [30, 31, 35]. Particularly, the ARDS is very heterogenous and PEEP is regularly applied to reduce inhomogeneity. However, Maiolo et al. reported variable effects of increased airway pressure and reported an increased inhomogeneity of 20% in mild ARDS while this effect was less pronounced or even negative in severe ARDS [39]. In agreement, further evidence suggests worsened outcome due to high pressure recruitment or high PEEP levels [37].

However, the threshold of optimized mechanical power is currently a matter of debate. Based on the previous study by Neto et al. [31] 25 J/min may discriminate between higher and lower lung damage, and iso-mechanical power results in similar degree of lung damage independent of whether the reason was high *V*_t_, respiratory rate or PEEP [28, 38]. This might be explained by an application of healthy animals but also due to exceeding the threshold causing maximal lung damage [28]. This hypothesis is in line with Cressoni et al. [39] reporting 12 J/min as threshold for mechanical power to induce VILI. Finally experimental and clinical trials to determine the optimal threshold for lung protective ventilation remain to be initiated.

### Should mechanical power be normalized in diseased lung tissue?

The inhomogeneity of the lung is associated with inhomogeneous distribution of forces and obviously of mechanical power which might subsequent result in the prior lung dependent reason for the progression of VILI [40]. Indeed, differences in elasticity were suggested to concentrate the applied forces by doubling [41]. For a determined mechanical power, intensity of ventilation is increased in lung tissue with fewer ventilated areas and at the interface between lung areas with different mechanical properties [15, 42]. Apart from the dependence of the lung surface, VILI might also be impaired by the open/closed interfaces which were associated with increased [(18F)FDG] uptake and subsequent increased proportion of lung condition severity [40, 43]. Finally, this suggests the total area of the well-inflated lung tissue as well as the inhomogeneous poorly inflated or uninflated lung tissue important potential parameter for normalization of mechanical power [41]. Recently, Coppola et al. reported that normalization of mechanical power as well as respiratory system compliance were prior compared to the mere values in prediction of mortality [33]. Normalization was based on a whole lung CT scan at 5 cm H_2_O of PEEP and performed after a recruitment maneuver and lung gas volume and amount of well-inflated tissue were computed [33, 41].

### Should ECMO be indicated to reduce intensity of ventilation?

Whether an earlier time point for initiation of ECMO therapy might an option, is currently under investigation (NCT04341285). Indeed, after implementation of the veno-venous EMCO therapy a so called “lung rest” with limited inspiratory plateau pressure (*P*_plat_) < 25 cm H_2_O which is highly recommended by the ELSO is mostly feasible [6]. Further reductions in the *P*_plat_ below 20 cm H_2_O were reported to be associated with fewer VILI and improved patient outcome [45, 47]. Ultra-protective- or even near-apneic –ventilation during ECMO support was reported to attenuate lung injury due to decreased *V*_*t*_ and driving pressure [45, 48]. Concordantly, Araos et al. [49] reported, that near-apneic ventilation caused histologic less lung injury compared to both, a non-protective and conventional ventilatory strategy in an experimental ARDS ECMO pig model. However, others detected no superiority of ultra-protective ventilation strategies during vvECMO [46, 50]. Particularly, near apneic ventilation strategies are associated with a risk of atelectasis with subsequent secondary infections and severe induction of ventilation/perfusion mismatch unless PEEP is appropriately increased to keep part of the lung open [51]. Additionally, ultra-protective ventilation requires deep sedation which is necessary to avoid patient—ventilator asynchrony. Which ventilation mode and subsequent lung unloading is necessary to secure recovery, healing and repair need to be determined by clinical trials.

An international multicenter prospective cohort study revealed, that most high ECMO volume centers prefer a “lung rest” pressure-controlled lung protective ventilation strategy [46]. In contrast, pre-ECMO ventilation intensity was less considered and mechanical power prior vvECMO implementation was with 26.1 ± 12.7 J/min particularly high [46]. This might be explained, that EMCO therapy is complex, labor-intensive, expensive and moreover a highly invasive procedure. Therefore some centers apply ECMO therapy as a kind of “last rescue” procedure for a severely hypoxemic population after several trials of optimal conventional ventilation, prone positioning and neuromuscular blockers have failed. However, increased duration of mechanical ventilation before ECMO therapy initiation might be associated with higher mortality rates after ECMO therapy. Recent logistic regression analyses revealed greater delay from endotracheal intubation to ECMO initiation is independently associated with 6 month mortality [17]. Otherwise, the mere duration of ventilation prior to ECMO implantation was not associated with increased mortality other reports [4]. More important seems to be the intensity of ventilation and particularly even short durations of high intensity ventilation might cause lung injury [32]. Subsequently, an individualized comprehensive twice daily analysis of ventilation parameters particularly mechanical power during the pre-ECMO period is highly recommended. If sufficient oxygenation and/or decarboxylation cannot be achieved with at least moderate ventilation intensity, a MP and/or DP limited ventilation strategy with subsequent lung unload by vvECMO should be critically evaluated.

### How could particularly MP be implemented during daily management?

The extended reference equation to calculate MP by Gattinoni et al. represents the most precise calculation [52, 53]. However, some variables like airway and tissue resistance or elastance of the respiratory system are complex to measure within the clinical routine setting [53]. Moreover, the application of this formula requires muscle relaxation and volume-controlled ventilation of the patients [53]. Meanwhile, several simplifications were developed for an application during the daily routine. For pressure-controlled ventilation two accurate equations were suggested, but require the knowledge of resistances and respiratory system compliance which are not determined within the daily routine [53–55]. However, Becher et al. recently suggested a simplified equation for MP calculation in pressure-controlled ventilated patients and Chiumello et al. reported bedside calculations of MP during volume- and pressure-controlled mechanical ventilation [55, 56]. Although this equations might be associated with a small bias of overestimation, it seems to be accurate and easy applicable during the daily routine [53]. Finally, the equations might also be applicable to spontaneous ventilation, but studies of accuracy and potential simplifications are currently lacking [57]. The current major limitation is, that airway pressure, flow and esophageal pressure are affected counter-directionally by the action of the ventilator and the respiratory muscles [57–59].

**Fig. 4:**
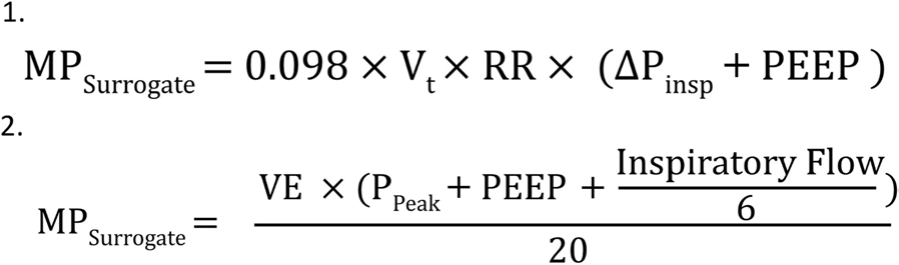
Simplified equations for (1) pressure-controlled ventilation [75] and (2) volume-controlled ventilation [76]. Both equations were approved to approximate the surrogate formulas well enough for application during daily routine management. [56]

Another disadvantageous of MP is the requirement for normalization on well aerated lung tissue. However, most vvECMO centers conduct CT scans as standardized diagnostic procedure. Alternately, normalization might be based on the lung compliance with a decreased predictive performance compared to the well-aerated tissue and the acceptance of a potential collinearity between MP and compliance [33]. A MP and DP-based ventilation strategy is shown in Fig. 2. Extended and simplified MP equations for volume—and pressure-controlled ventilation are shown in Figs. 3 and 4.

### Which open questions need to be addressed in large clinical trials?

The relevance of DP and MP for vvECMO indication is just beginning. The key question clinical trials should address is the time point at which a lung unload and vvECMO support is necessary to avoid further ventilator-induced lung injury e.g., is the prize of sufficient oxygenation reasonable? With other words, threshold levels for pre-defined equations in dependence of ventilation strategy have to be determined. If adaptions of the ventilator strategy do not result in decreased MP, vvECMO implantation should be re-checked critically. However, the relative relevance of the MP components on VILI are currently not completely clear. Indeed, mathematically, MP increases with the exponent of 2 of Vt, the exponent of 1.4 of the RR and the exponent of 1 of PEEP. Whether an optimal composition of these parameters might be preferable to reduce VILI should be evaluated during the pre-ECMO ventilation strategy [60, 61].

However, in the case of ARDS, the development of innovative trial design is generally associated with several challenges. First, molecular biology of ARDS is very heterogeneous between patients with considerably differences in biomarkers of key pathways including inflammation, coagulation, and alveolar epithelial injury [62–64]. Therefore, a rigorously phenotyped patient large collective seems to be essential [62, 64]. Recently, the development of assay platforms based on protein-based enrichments strategy was suggested by Beitler et al. which might be most efficient to overcome this task [62, 65].

Second, outcome definition of ARDS in clinical trials is challenging [62]. Although mortality is frequently used in clinical trials as endpoint, ARDS-related risk of mortality differs considerably between patients and diseases. Moreover, endpoints other than mortality have to address death as a competing risk. However, this might be resolved by the application of a ranked composite score which compares each patient to all other patients encompassed in the study by vital status and subsequently, only if both patient in pair survive by the second to be analyzed outcome parameter. [62, 66, 67]

Third, VILI-related lung injury and effect of different ventilator strategies on VILI are not simply detected at bedside and separated form disease-related lung injury [68]. Frequently, global respiratory mechanics and the degree of ventilator support required were applied to characterize ARDS suffering patients [62]. However, particularly to separate disease from ventilator-related lung injury and to evaluate thresholds of DP and MP for ECMO indication, a standardized preclinical animal approach with an option of post-mortal lung injury characterization and associated biomarker and proteome analysis seems a reasonable approach. However, particularly lung compliance, airflow and VILI-induced inflammation seem to be highly species dependent [69]. In contrast to humans, most of the elastic recoil measured in intact mice can be attributed to the lung as chest wall and further thoracic structures are very compliant [69, 70]. Moreover, species dependent differences in inflammation and innate immunity were reported [71]. Exemplarily, Toll like receptor 4 from humans and mice recognize different lipopolysaccharide [72] and mice lack the CXCL8 gene coding IL8 [73, 74], a potent neutrophil chemotactic factor with a key role in the pathogenesis of ARDS. Although some this limitations might be overcome by the application of a large animal model, currently none of these models adequately reproduce the full characteristics of human ARDS. Therefore, subsequent bench to bedside approaches are indispensable.

## Conclusion

The most challenging issues in ARDS patient treatment are the heterogeneity of the population and the continuously changing pulmonary circumstances. Therefore, an individualized patient and continuously adapted ventilation strategy is highly recommended (Fig. 2). Although much work has to be done to evaluate these strategies and thresholds, current evidence suggests, that driving pressure guided ventilation might decrease mechanical power and decreased mechanical power seems to be associated with decreased VILI. In particular, the mechanical power normalized to well inflated tissue and to respiratory system compliance were reported being independently associated with mortality of ARDS suffering patients [33]. The landmark paper by Umer et al. [32] analyzed 13,939 patients and suggested that higher intensities of mechanical ventilation reflected by driving pressure and mechanical power was harmful, even for short durations.  Whether finally limited exposure of driving pressure and mechanical power, which might subsequent result in earlier initiation of extracorporeal support (ECMO, ECCO2R) improve ARDS patients` outcome has to be investigate by randomized controlled trials. But until the results are available, we highly recommend to implement driving pressure and mechanical power during daily treatment of ARDS patients and subsequent ECMO therapy.

## Data Availability

Not applicable.
